# Silymarin in Combination with Vitamin C, Vitamin E, Coenzyme Q10 and Selenomethionine to Improve Liver Enzymes and Blood Lipid Profile in NAFLD Patients

**DOI:** 10.3390/medicina56100544

**Published:** 2020-10-17

**Authors:** Annalisa Curcio, Adriana Romano, Simona Cuozzo, Antonio Di Nicola, Orazio Grassi, Donatella Schiaroli, Giuseppe Fabrizio Nocera, Michele Pironti

**Affiliations:** 1 Medical Department, Aqma Italia S.p.A., 20123 Milan, Italy; michele.pironti@aqma.it; 2 Medical Department, Merqurio Pharma S.r.l., 80138 Naples, Italy; adriana.romano@merqurio.it (A.R.); simona.cuozzo@merqurio.it (S.C.); 3 Infectious Disease Department, Cardarelli Public Hospital, 86100 Campobasso, Italy; antonio.dinicola@libero.it (A.D.N.); orazio.grassi@libero.it (O.G.); 4 General Practitioner, 91100 Trapani, Italy; donatella.schiaroli@gmail.com; 5 General Practitioner, 59100 Prato, Italy; giuseppe.nocera@gmail.com

**Keywords:** non-alcoholic fatty liver disease (NAFLD), silymarin, coenzyme Q10, vitamin C, vitamin E, selenomethionine

## Abstract

*Background and Objectives:* Non-Alcoholic Fatty Liver Disease (NAFLD) is an emerging cause of hepatopathy that is showing an increasing trend and where the recommendations of lifestyle modification are often not sufficient. The aim of this study is to evaluate the efficacy and tolerability profile of the association of silymarin, vitamin C, vitamin E, coenzyme Q10 and selenomethionine (Medronys epato^®^) by analyzing liver enzymes, along with the lipidic profile, as markers of liver function, and ultrasound results in NAFLD patients. *Materials and Methods:* This study enrolled 81 patients with mild to severe NAFLD, divided into two groups: Group A (*N* = 41) received two capsules a day of silymarin, vitamin C, vitamin E, coenzyme Q10 and selenomethionine (Medronys epato^®^), and Group B (*N* = 40) received only recommendations for lifestyle modification including hypocaloric diet, physical exercise and encouragement for weight loss. Patients have been evaluated at three timepoints: baseline (T0), after 45 days of treatment (T1) and after 90 days of treatment (T2), by collecting blood parameters of alanine aminotransferase (ALT), aspartate aminotransferase (AST), alkaline phosphatase (ALP), gamma-glutamyl transferase (GGT) and the lipid blood profile. Ultrasonographic results have been analyzed at T0 and T2, along with the tolerability profile and side effects, registered at time T2. *Results:* The administration of the association of silymarin, vitamin C, vitamin E, coenzyme Q10 and selenomethionine (Medronys epato^®^) was effective since it showed a significant reduction of the evaluated parameters of ALT, AST, ALP and GGT, a significant improvement of lipid parameters, evaluated as markers of liver function, and improvements of ultrasonographic results. The use of this formulation at the dosage of two capsules a day has been well tolerated and no adverse events have been reported during study period of three months. *Conclusions:* The administration of the association of silymarin, vitamin C, vitamin E, coenzyme Q10 and selenomethionine (Medronys epato^®^) was effective and well tolerated in the improvement of hepatic function of NAFLD patients.

## 1. Introduction

Non-Alcoholic Fatty Liver Disease (NAFLD) is an emerging cause of hepatopathy that has been showing an increasing trend in the last two decades, with an estimated doubling of prevalence, compared to the prevalence of other chronic liver diseases that have shown a stationary incidence among the past years [1].

NAFLD is a condition characterized by an accumulation of lipids, mainly triglycerides, also defined as steatosis, in the hepatocytes of the liver parenchyma, which is responsible for the onset of hepatic damage, causing persistent increased levels of hepatic enzymes. A simple steatosis can be considered as a reversible condition that may not lead to complications; however, a significant percentage of NAFLD patients (which consist of 59.10% NAFLD patients who had liver biopsy for a “clinical indication,” and of a percentage ranging from 6.67–29.85% of NAFLD patients who had a liver biopsy without a specific “clinical indication”) can reach out to a disease progression, with hepatic injury, inflammation and tissue necrosis, typical of the more serious disease Non-Alcoholic SteatoHepatitis (NASH) [1,2]. NASH shows histological aspects of necroinflammation and hepatocytes degeneration with ballooned appearance, along with fibrosis. The evolution of NAFLD in NASH can lead to more severe complications such as cirrhosis, liver failure and hepatocellular carcinoma [1,3]. For this reason, it is crucial for physicians to try to avoid the degeneration of NAFLD to more severe pathologies.

Pathogenesis of NAFLD involves complex mechanisms, which, to simplify, can be divided into two types: the metabolic pathways, involving hepatic lipid accumulation, sedentary lifestyle, high fat diet, obesity and insulin resistance, and the inflammatory pathways with associated fibrogenesis. The metabolic pathways lead to consider NAFLD as the hepatic manifestation of the metabolic syndrome, which includes obesity, dyslipidaemia, hypertension and type 2 diabetes mellitus (T2DM). The inflammatory pathways involve oxidative stress, which in turn is responsible for increased lipid peroxidation, high reactive oxygen species production within hepatocytes, mitochondrial dysfunction and lipotoxicity [3,4].

Diagnosis of NAFLD, as recommended by World Gastroenterology Organization, contemplates a hierarchical resource-sensitive approach, in order to limit the use of invasive and expensive exams, like biopsies, and to prefer non-invasive and cheaper tools, like ultrasonography, serum biomarkers and liver enzymes, with the exclusion of other liver diseases. However, it is recognized that liver biopsy is required for NAFLD/NASH confirmation and staging [3,5].

More common markers of liver injury are alanine aminotransferase (ALT), aspartate aminotransferase (AST), alkaline phosphatase (ALP), gamma-glutamyl transferase (GGT) and bilirubin [1,6]. In daily clinical practice, measurement of above-mentioned liver enzymes is often associated with the blood lipid profile and insulin resistance measurement, which are used to evaluate and diagnose NAFLD. Several studies confirm that serum biomarkers, such as triglycerides, total cholesterol and low-density lipoprotein (LDL) cholesterol, correlate with NAFLD severity [4,7,8,9,10].

Actually, there are no specific drugs approved for the NAFLD/NASH therapy, and pharmacological treatments specifically aimed at improving liver disease are reserved to advanced-stage NASH patients with fibrosis. In this case, several treatments are under study, including pioglitazone and vitamin E, which can be considered as treatment options for biopsy-proven NASH patients with and without diabetes, respectively, after discussion about the risk/benefit ratio with every patient [2,11].

On the other hand, the only intervention strongly recommended for both NAFLD and NASH patients is lifestyle modification consisting of diet, exercise and weight loss, together with the management of metabolic comorbidities, like obesity, dyslipidemias, diabetes and insulin resistance [1,2,3,11]. Many patients have difficulties achieving weight loss and, in general, modifying their lifestyle, so the use of natural supplements with antioxidant and/or hepatoprotective activity can be a valid support both in NAFLD management and in the prevention of NAFLD degeneration in fibrosis and NASH. Further, many NAFLD patients are of normal weight, confirming the complexity of the pathogenetic profile of NAFLD [3,11].

Among the active substances used for the relief of NAFLD symptoms, an important one is *Silybum marianum*, also named milk thistle, which has been known for a long time for its activity in liver diseases. The active extract of *Silybum marianum* consists approximately of 70–80% of silymarin with a mixture of flavonolignans, including silybin, a component with major hepatoprotective properties. Silymarin employs multiple mechanisms as a hepatoprotective agent, and between the most important are antioxidant activity and cell-regenerating functions as a result of increased protein synthesis, along with anti-inflammatory and antifibrotic properties. Overall, the effect of silymarin can be summarized in four activities:

1. Ability to counteract lipid peroxidation by means of free radical scavenging and increasing cellular glutathione (GSH) content.
2. Action on membrane permeability, which allows an increase of membrane stability in the presence of xenobiotic damage.
3. Regulation of nuclear expression in a steroid-like manner.
4. Inhibition of conversion from stellate hepatocytes into myofibroblasts, leading to collagen fibers production and depositing, with increase of fibrosis and tissue degeneration in more severe hepatic diseases [12,13].

Several studies analyzed the effect of silymarin in NAFLD patients, showing its efficacy in the reduction of liver enzymes, serum biomarkers and ultrasonography results [14,15,16,17,18,19,20,21,22,23]. Furtherly, significant results in the treatment of NAFLD have been reported in other studies, where active substances, like vitamin E, vitamin C, coenzyme Q10 and selenium, have been used in NAFLD patients for their antioxidant and anti-inflammatory proprieties. These compounds showed their effect on liver enzymes, inflammatory pathways and the improvement of health status of NAFLD patients, and have been in turn used in association with silymarin in order to improve its efficacy [16,17,19,20,23,24,25,26].

A previous study showed the efficacy of the association of silymarin, vitamin C, vitamin E, coenzyme Q10 and selenomethionine in improving blood parameters (ALT, AST, ALP, GGT and ferritin), symptoms and ultrasonography in patients with NAFLD [24].

The aim of this study is to evaluate the efficacy and tolerability profile of the association of silymarin, vitamin C, vitamin E, coenzyme Q10 and selenomethionine (Medronys epato^®^), by analyzing liver enzymes, along with the lipidic profile, as markers of liver function, and ultrasound results in NAFLD patients.

## 2. Materials and Methods

This is an observational study that enrolled 81 patients (50 male, 31 female), mean age: 62.2 ± 13.9 years, with mild to severe NAFLD; in particular, in 31% (*N* = 25) of patients, NAFLD was mild, in 46% (*N* = 37) it was moderate and in 23% (*N* = 23) it was severe. NAFLD diagnosis was confirmed by clinical examination, liver enzyme and ultrasonography. Exclusion criteria were: any chronic and acute liver disease including viral hepatitis C, hepatitis B, chronic or acute kidney disease, alcohol consumption (men, more than 20 g/day, and women, more than 10 g/day), pregnancy, taking medications affecting the liver such as steroids, amiodarone and tamoxifen, and patients with proven hemochromatosis.

The patients were randomly divided in two groups: Group A (*N* = 41) was the treatment group and received 2 capsules a day of silymarin, vitamin C, vitamin E, coenzyme Q10 and selenomethionine (Medronys epato^®^, Aqma Italia S.p.A., Milan, Italy), corresponding to 350 mg of Silybum marianum dry extract titrated to 80% in silymarin (280 mg), 120 mg of vitamin C, 40 mg of vitamin E, 20 mg of coenzyme Q10 and 83 µg of selenium. Group B (*N* = 40) was the control group and did not receive treatment, but only recommendations for lifestyle modification including hypocaloric diet, physical exercise and encouragement to weight loss. The same recommendations have been provided also to Group A patients. All subjects gave their informed consent for inclusion before they participated in the study. The study was conducted in accordance with the Declaration of Helsinki, and the protocol was approved by the Ethics Committee of Aqma Italia S.p.A. in Naples (protocol n. 20190001802 on 18 February 2019).

The study period was 90 days, and patients have been evaluated at 3 timepoints: baseline (T0), after 45 days of treatment (T1) and after 90 days of treatment (T2).

At each timepoint, the following parameters have been collected: blood parameters of ALT, AST, ALP and GGT, and the lipid blood profile, including total cholesterol (TC), Low-density lipoprotein cholesterol (LDL-C), High-density lipoprotein cholesterol (HDL-C) and triglycerides (TG). Blood samples were collected after 12–14 h of fasting by an antecubital venous puncture. Serum samples were obtained by centrifugation. Serum levels of AST, ALT, GGT, ALP and the lipid profile were directly measured using standard automated laboratory methods on Cobas 6000 (Roche, Rotkreuz, Switzerland) by using the relative kits according to the manufacturer’s instructions.

Further, ultrasonographic results have been analyzed at baseline and at time T2, along with the tolerability profile and side effects, registered at time T2.

Liver ultrasonography evaluations have been performed by using an HDI 5000 Ultrasound System (Philips Medical Systems, Bothell, WA, USA). Evaluated parameters were: liver brightness compared with that of the kidney, attenuation of the sonographic beam and blurring of vessels [27]. By analyzing these parameters, two experienced sonographers assigned a degree of liver steatosis of “no steatosis,” “mild,” “moderate” or “severe” to each patient at baseline and at T2. When the initial degree did not change, the result has been classified as “no improvement;” when the initial score decreased from “severe” to “moderate” or from “moderate” to “mild,” the result has been classified as “mild improvement;” when the initial score decreased from “severe” to “mild” or from “moderate” to “no steatosis,” the result has been classified as “large improvement.”

Data recorded were normally distributed and expressed as means with standard deviations (SD).

Preliminary statistical analysis was performed using Paired *t*-test with the SigmaStat v. 3.5 (San Jose, CA, USA) analysis program by comparing all serum parameters included in the study recorded at baseline, after 45 days and after 90 days. The one-way analysis of variance (ANOVA) test is used to test for differences among at least three groups. When there are only two means to compare, the *t*-test and the F-test are equivalent. To confirm the preliminary data, a second statistical analysis was performed using the one-way ANOVA test (Holm-Sidak test) to compare the variation between the treatment groups. The differences were considered significant when *p* < 0.05.

## 3. Results

### 3.1. Baseline Patients Characteristics

The study enrolled 81 patients, with newly diagnosed NAFLD and of varying severity, which has been evaluated by blood parameter of ALT, AST, ALP and GGT, along with liver ultrasonography examination, performed by physicians both to confirm liver steatosis diagnosis and to evaluate steatosis degree.

The mean age was 56.9 (+14.2) years in Group A patients and 67.6 (+11.3) years in Group B patients (Table 1). The authors did not investigate the eventual implications of the age on results, as they consider this event not relevant.

In the treatment group (Group A), the steatosis degree was the following: 29.3% (*N* = 12) patients with mild steatosis, 46.3% (*N* = 19) patients with moderate steatosis and 24.4% (*N* = 10) with severe steatosis. In the control group (Group B), the steatosis degree was the following: 32.5% (*N* = 13) patients with mild steatosis, 45% (*N* = 18) patients with moderate steatosis and 22.5% (*N* = 9) with severe steatosis.

In Group A and B, patients with comorbidities were 61% and 70%, respectively. The most frequent comorbidities were hypertension, dyslipidemia and type 2 diabetes mellitus.

Liver biopsy was not performed in order to limit and/or avoid invasive procedures. Demographic characteristics of the study population are summarized in Table 1.

### 3.2. Efficacy Results

#### 3.2.1. Liver Enzymes Results

Group A patients showed a statistically significant reduction of all liver enzymes evaluated (Table 2). Achieved results displayed that Group A patients after 45 days already reported a significant reduction of the evaluated parameters, showing the rapidity of the action of the treatment, with recovering of normal values of liver enzymes after 90 days of treatment.

Group B patients showed a statistically significant reduction only for ALT and AST parameters (Table 2).

#### 3.2.2. Lipid Profile Results

The analysis of the lipid profile in Group A patients showed a reduction of TC, C-LDL and TG, along with an increased level of HDL-C, all changes were statistically significant (Table 3). Also, these results showed the rapidity of the action of the treatment in improving the lipid profile already after 45 days, with further improvement after 90 days.

In Group B patients (control group), a statistically significant reduction of TC and LDL-C parameters occurred (Table 3). Further, changes in HDL-C and TG were not significant.

#### 3.2.3. Ultrasonographic Results

Ultrasonographic results showed that, in Group A patients, a large improvement occurred in 51.3% (*N* = 21) of patients, a mild improvement in 34.1% (*N* = 14) of patients and no improvement in 14.6% (*N* = 6) of patients. In Group B patients, a large improvement occurred in 15% (*N* = 6) of patients, a mild improvement in 32.5% (*N* = 13) of patients and no improvement in 52.5% (*N* = 21) of patients.

### 3.3. Safety Results

Oral treatment with 2 capsules a day of silymarin, vitamin C, vitamin E, coenzyme Q10 and selenomethionine (Medronys epato^®^) has been well tolerated in patients with NAFLD enrolled in this study. No adverse events have been reported during the study period in both groups of patients.

## 4. Discussion

This study evaluated the efficacy and tolerability profile of the association of silymarin, vitamin C, vitamin E, coenzyme Q10 and selenomethionine (Medronys epato^®^) administered at dosage of 2 capsules a day in patients with NAFLD. This supplement has been formulated for a rapid hepatic recovery in chronic liver diseases of different etiology, by repristinating physiological functions of the hepatocytes, and consequently reducing liver enzymes levels and improving the lipid profile [12,13,14,15,16,17,18,19,20,21,22,23,24,25,26].

Several studies demonstrated the efficacy of silymarin in NAFLD patients by the reduction of liver enzymes, serum biomarkers and ultrasonographic results [14,15,16,17,18,19,20,21,22,23,24].

Several other substances with antioxidant and anti-inflammatory activity, like vitamin E, vitamin C, coenzyme Q10 and selenium, have been studied for their effect in the improvement of steatosis and liver enzymes in NAFLD patients, and showed their efficacy in the reduction of transaminases levels and in improving the fatty liver index. Often, these substances have been administered in combination with silymarin in order to improve its efficacy [16,17,19,20,23,24,25,26]. The formulation in study, made by the association of silymarin, vitamin C, vitamin E, coenzyme Q10 and selenomethionine (Medronys epato^®^), was designed to exploit the synergic effect of the single active ingredients added [24].

This formulation has been previously studied at dosage of 1 capsule a day in patients with NAFLD, showing efficacy in the improvement of liver enzymes and symptomatology, along with a good tolerability profile [24]. In this study, we explored the use of doubling the dosage of this formulation to evaluate if the effect is dose-dependent along with confirming its safety and evaluating efficacy results as improvement in both the liver enzymes and the lipid profile in NAFLD patients.

The results of this study showed the efficacy of the administration of the association of silymarin, vitamin C, vitamin E, coenzyme Q10 and selenomethionine (Medronys epato^®^), since in Group A patients, a significant reduction of evaluated parameters of ALT, AST, ALP and GGT already occurred after 45 days of treatment, which become more important after 90 days of treatment, when liver enzymes values showed a return to values generally recognized as normal.

Dyslipidemia is known as a risk factor for NAFLD, and several studies confirmed that in NAFLD patients, there is a relationship between steatosis degree and lipid profile [7,8,9,10]. Therefore, it is of crucial importance to assess the effect of an intervention on lipidic parameters, and in this study, Group A patients showed a significant improvement of lipid parameters, with reduction of TC, C-LDL and TG, and an increase of HDL-C levels, with a potential effect also in the cardiovascular outcomes of such patients.

Ultrasound analyses confirmed results obtained with the liver enzymes and lipid profile by showing imaging improvements and a major percentage of patients with more important improvements in Group A patients.

The effect of the association studied after only 45 days of treatment allows us to assume that the synergic effect of the active compounds in the formulation improves the rapidity of the action of this intervention.

Group B patients that received only recommendations for lifestyle modification with encouragements to weight loss by hypocaloric diet and physical activity also reported a mild improvement of liver enzymes, lipid profile and ultrasonography, but in some cases, it was not significant. This can confirm that often, in real life, the intervention with only lifestyle modification is not enough. We can suppose that many patients can be not compliant with lifestyle modification and can have difficulties in achieving weight loss and doing regular physical exercise. In this context, the use of a natural supplement, such as the association of silymarin, vitamin C, vitamin E, coenzyme Q10 and selenomethionine (Medronys epato^®^), can be a safe and useful tool to provide a support to NAFLD patients not compliant to lifestyle modification recommendations [3,11].

Despite the encouraging results, this study has some limitations that can derive from the limited number of patients enrolled and from the single judgement of the imaging improvement derived from the ultrasound evaluation of every physician that participated to this study, along with the absence of biopsy examination of patients, which has been avoided to limit the use of invasive procedures. However, in the future, it will be interesting to evaluate the effect of the association of silymarin, vitamin C, vitamin E, coenzyme Q10 and selenomethionine on fibrosis markers and to investigate whether this supplementation can be useful in more severe conditions, like liver fibrosis and NASH.

Further research will be directed to the evaluation of the use of the administration of the association of silymarin, vitamin C, vitamin E, coenzyme Q10 and selenomethionine (Medronys epato^®^) in different settings and patient types, also considering patients with other chronic liver diseases, such as viral hepatitis C and B, or alcohol abuse patients.

## 5. Conclusions

In conclusion, this study showed the efficacy of the administration of the association of silymarin, vitamin C, vitamin E, coenzyme Q10 and selenomethionine (Medronys epato^®^) in the improvement of hepatic function of NAFLD patients, as evaluated by a significant reduction of liver enzymes (ALT, AST, ALP and GGT), by a significant improvement of lipid parameters (TC, C-LDL, HDL-C and TG) analyzed as markers of liver function and by the improvements of liver ultrasonography. Further, the use of this formulation at the dosage of 2 capsules a day has been well tolerated and no adverse events have been reported during a study period of 3 months.

## Figures and Tables

**Table 1:** Baseline characteristics of Group A (treatment group) and Group B (control group).

Variable	Group A	Group B
Male/female, N	25/16	25/15
Age (years), mean (SD)	56.9 (14.2)	67.6 (11.3)
Steatosis degree (%)	Mild 29.3	Mild 32.5
	Moderate 46.3	Moderate 45
	Severe 24.4	Severe 22.5
Comorbidities (%)	61	70

**Table 2:** Changes in liver enzymes in Group A (treatment group) and Group B (control group), at T0 (baseline), T1 (45 days) and T2 (90 days).

Liver Enzymes IU/L, Mean (±SD)	Group A				Group B			
	T0	T1	T2	*p* Value	T0	T1	T2	*p* Value
**ALT**	81.00 (22.46)	55.69 (18.02)	36.54 (9.85)	**<0.001**	90.11 (39.28)	81.33 (35.80)	74.00 (32.58)	**<0.001**
**AST**	71.85 (23.88)	41.23 (13.47)	29.85 (10.53)	**<0.001**	88.44 (32.89)	78.33 (31.86)	73.44 (32.46)	**<0.001**
**ALP**	118.46 (41.12)	93.21 (45.11)	82.15 (44.10)	**<0.001**	95.03 (57.12)	90.44 (55.16)	90.37 (56.51)	0.351
**GGT**	78.46 (24.37)	43.54 (15.54)	30.08 (9.02)	**<0.001**	80.67 (25.14)	76.56 (20.28)	75.56 (18.23)	0.095

SD = standard deviation; ALT = alanine aminotransferase, AST = aspartate aminotransferase, ALP = alkaline phosphatase, GGT = gamma-glutamyl transferase. Statistically significant results are reported in bold.

**Table 3:** Changes in lipid profile in Group A (treatment group) and Group B (control group), at T0 (baseline), T1 (45 days) and T2 (90 days).

Lipid Parameters mg/dL, Mean (±SD)	Group A				Group B			
	T0	T1	T2	*p* Value	T0	T1	T2	*p* Value
**TC**	217.77 (37.26)	199.00 (30.53)	182.31 (25.89)	**<0.001**	223.33 (28.25)	217.44 (27.04)	211.00 (6.31)	**<0.001**
**LDL-C**	140.00 (21.56)	126.85 (18.16)	113.31 (16.68)	**<0.001**	154.00 (26.13)	149.33 (24.57)	142.56 (22.93)	**<0.001**
**HDL-C**	54.08 (11.88)	58.00 (9.55)	60.46 (8.01)	**<0.001**	45.33 (7.28)	48.11 (5.79)	49.33 (5.22)	0.039
**TG**	199.85 (98.82)	166.54 (73.83)	139.38 (54.78)	**<0.001**	199.56 (68.46)	192.67 (61.80)	188.89 (60.15)	0.023

SD = standard deviation; TC = total cholesterol, LDL-C= Low-density lipoprotein cholesterol, HDL-C = High-density lipoprotein cholesterol, TG = triglycerides. Statistically significant results are reported in bold.
